# Characterization of the GDP-D-Mannose Biosynthesis Pathway in *Coxiella burnetii*: The Initial Steps for GDP-β-D-Virenose Biosynthesis

**DOI:** 10.1371/journal.pone.0025514

**Published:** 2011-10-31

**Authors:** Craig T. Narasaki, Katja Mertens, James E. Samuel

**Affiliations:** Texas A&M University Health Science Center, College of Medicine, College Station, Texas, United States of America; Monash University, Australia

## Abstract

*Coxiella burnetii*, the etiologic agent of human Q fever, is a Gram-negative and naturally obligate intracellular bacterium. The O-specific polysaccharide chain (O-PS) of the lipopolysaccharide (LPS) of *C. burnetii* is considered a heteropolymer of the two unusual sugars β-D-virenose and dihydrohydroxystreptose and mannose. We hypothesize that GDP-D-mannose is a metabolic intermediate to GDP-β-D-virenose. GDP-D-mannose is synthesized from fructose-6-phosphate in 3 successive reactions; Isomerization to mannose-6-phosphate catalyzed by a phosphomannose isomerase (PMI), followed by conversion to mannose-1-phosphate mediated by a phosphomannomutase (PMM) and addition of GDP by a GDP-mannose pyrophosphorylase (GMP). GDP-D-mannose is then likely converted to GDP-6-deoxy-D-*lyxo*-hex-4-ulopyranose (GDP-Sug), a virenose intermediate, by a GDP-mannose-4,6-dehydratase (GMD). To test the validity of this pathway in *C. burnetii*, three open reading frames (CBU0671, CBU0294 and CBU0689) annotated as bifunctional type II PMI, as PMM or GMD were functionally characterized by complementation of corresponding *E. coli* mutant strains and in enzymatic assays. CBU0671, failed to complement an *Escherichia coli manA* (PMM) mutant strain. However, complementation of an *E. coli manC* (GMP) mutant strain restored capsular polysaccharide biosynthesis. CBU0294 complemented a *Pseudomonas aeruginosa algC* (GMP) mutant strain and showed phosphoglucomutase activity (PGM) in a *pgm E. coli* mutant strain. Despite the inability to complement a *manA* mutant, recombinant *C. burnetii* PMI protein showed PMM enzymatic activity in biochemical assays. CBU0689 showed dehydratase activity and determined kinetic parameters were consistent with previously reported data from other organisms. These results show the biological function of three *C. burnetii* LPS biosynthesis enzymes required for the formation of GDP-D-mannose and GDP-Sug. A fundamental understanding of *C. burnetii* genes that encode PMI, PMM and GMP is critical to fully understand the biosynthesic pathway of GDP-β-D-virenose and LPS structure in *C. burnetii*.

## Introduction

Lipopolysaccharide (LPS) is a complex molecule and represents the major component of the outer leaflet of the outer membrane of Gram-negative bacteria. The LPS molecule consists of three structural domains: (1) lipid A, which represent the hydrophobic anchor of the LPS molecule and is responsible for the endotoxic properties, (2) a short non-repeating inner and outer core oligosaccharide, which is attached to lipid A and extends outwardly and (3) the O-specific polysaccharide chain (O-PS), which is composed of repeating sugar units and determines the serological heterogeneity among bacterial isolates. The primary function of LPS is to serve as a permeability barrier against external agents such as hydrophobic antibiotics and to maintain the structural integrity of the Gram-negative cell wall [1].

*C. burnetii*, a Gram-negative small pleomorphic coccobacillus, is the causative agent of the zoonosis Q fever. Q fever manifests in humans generally as an acute, debilitating flu-like illness or less common as chronic Q fever, which develops mainly as endocarditis or hepatitis. C. *burnetii* is a naturally obligate intracellular bacterium and so far no method for generation of specific mutants has been established. *C. burnetii* is considered a potential biological weapon because it consistently causes disability, can be manufactured on a large scale, remains stable under various conditions and can be efficiently disseminated [2]. The U.S. Centers for Disease recently designated *C. burnetii* as a category B bioterrorism agent. There is no licensed vaccine for *C. burnetii* infection in the U.S. because of adverse reactions to killed whole cell vaccination. Therefore, the understanding of *C. burnetii* physiology and vaccine development remains an important public health and U.S. national security objective [3].

Upon serial passage in an immune-incompetent host, virulent *C. burnetii* undergoes a shortening of its LPS, traditionally referred to as phase variation in *Enterobacteriaceae*. Phase variation of *C. burnetii* is characterized by a non-reversible switch from virulent phase I smooth LPS (S-LPS), which has a full length O-polysaccharide (O-PS) chain to an avirulent phase II rough-LPS (R-LPS). The R-LPS variant is missing the O-PS chain and unknown sugar residues located within the outer core oligosaccharide [4]. Previous studies showed that no significant loss of protein content on the surface of *C. burnetii* occurred during phase variation and the only characterized difference between virulent phase I and avirulent phase II isolates is LPS [5], [6], [7], [8]. Furthermore, vaccine studies showed that BALBc mice vaccinated with formalin killed whole cell phase I bacteria were protected from *C. burnetii* challenge while mice vaccinated with whole cell phase II bacteria were not protected [8]. These studies highlight the importance of *C. burnetii* LPS.

Structural and compositional studies revealed several unique characteristics of the LPS molecule of *C. burnetii* LPS [9], [10], [11], [12], [13], [14], [15], [16], [17]. The lipid A moiety contains a typical 1 and 4′ phosphorylated, β-(1?6)-linked D-glucosamine (GlcN) disaccharide backbone, but is tetraacylated [17]. The inner core oligosaccharide is composed of D-mannose (D-Man), D-*glycero*-D-*manno*-heptose (D,D-Hep) and 3-deoxy-α-D-*manno*-oct-2-ulopyranoside (Kdo), in the molar ratio 2∶2∶3, comparable to the enterobacterial inner core region [13]. However, composition and structure of the O-PS chain is not entirely resolved. Two unique branched sugar residues, β-D-virenose (6-deoxy-3-C-methyl-D-gulose) and L-dihydrohydroxystreptose (3-C-(hydroxymethyl)-L-lyxose), were detected in heteropolysaccharide fractions of isolated LPS [18], [19]. To our knowledge, virenose is not found on the surface structures of any other microorganism except *C. burnetii* LPS. Subsequent studies resolved the structure of virenose, while linkage and chemical compositional analysis indicated that *C. burnetii* O-PS is likely a heteropolymer of 1?4 linked β-D-virenose, dihydrohydroxystreptose and mannose [19], [20]. These findings are consistent with the observation that ABC transporter encoding genes *wzm* (CBU0703) and *wzt* (CBU0704) are located in a genomic region associated with O-PS synthesis [21]. ABC transporters are usually involved in biosynthesis of homopolymeric or small repeating units containing herteropolymeric O-PS [1].

**Figure 1:**
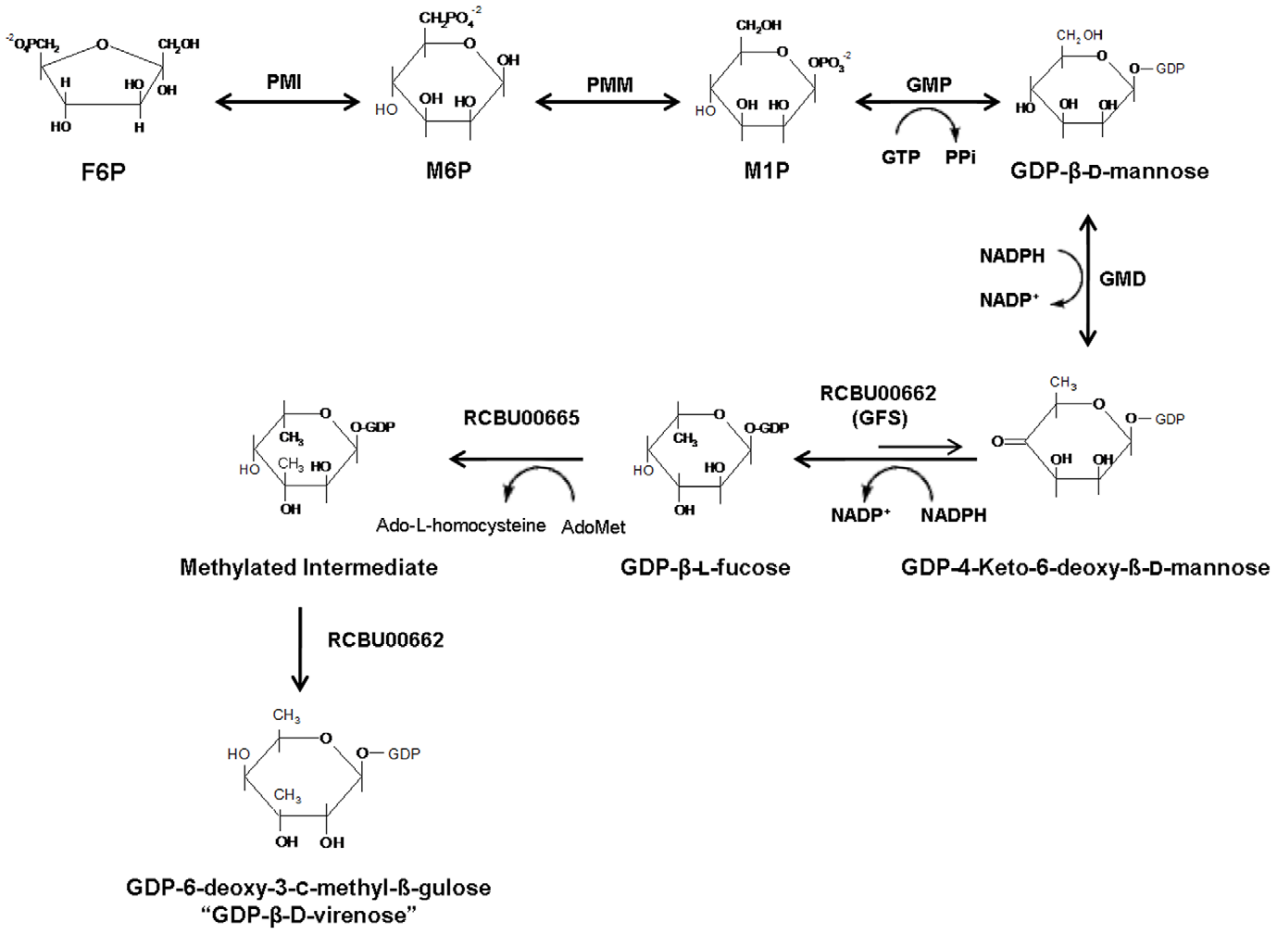
Putative GDP-β-D-virenose biosynthesis pathway. 1. F6P, fructose-6-phosphate; PMI, phosphomannose isomerase 2. M6P, mannose-6-phosphate; PMM, phosphomannomutase 3. M1P, mannose-1-phosphate, GMP, GDP-mannose pyrophosphorylase 4. GMD, GDP-mannose 4,6-dehydratase; NADP^+^ nicotinamide adenine dinucleotide phosphate 5. GFS, fucose synthase 6. Ado-Met, S-adenosyl methionine.

Phase variation in *C. burnetii* is accompanied by the deletion of a large chromosomal fragment which contains glycosyl transferases and sugar processing genes required to complete β-D-virenose biosynthesis, O-PS chain elongation and inner membrane transport [21], [22]. This deletion is likely the O-PS operon and is responsible for the loss of O-PS in the *C. burnetii* Nine Mile strain RSA439 [21]. Based on the structure of β-D-virenose and the genes located within the deleted region of the *C. burnetii* phase II variant, the in Figure 1 presented GDP-β-D-virenose biosynthesis pathway is proposed. The aim of this study was to demonstrate the biological significance of three *C. burnetii* enzymes for the biosynthesis of GDP-D-mannose and examine the initial steps of GDP-β-D-virenose biosynthesis. The presented data provide fundamental knowledge necessary to further characterize the formation of GDP-β-D-virenose, a novel saccharide, and may help develop potential vaccine candidates such as *in vivo* and *in vitro* generated glycoconjugates.

## Results

Bioinformatic analysis was carried out on the *C. burnetii* enzymes predicted to be responsible for GDP-D-mannose biosynthesis (Table 1). Amino acid sequence alignments indicated that each enzyme had a high degree of similarity to characterized GDP-mannose biosynthesis enzymes. *C. burnetii* CBU0671 has the bioinformatic signatures of a type II phosphomannose isomerase (PMI), a small but growing class of PMIs identified in Gram-negative bacteria [23]. Type II PMIs are bifunctional enzymes that catalyze the isomerisation of fructose-6-phosphate to mannose-6-phosphate and the transfer of GDP to D-mannose-1-phosphate to form GDP-D-mannose [24]. However, *C. burnetii* CBU0671 appeared to be unrelated to a type I PMI from *E. coli* EDL933, but contains the conserved PMI active site, which is characteristic of the type II PMIs [23]. *C. burnetii* CBU0294 is predicted to catalyze the second step in the GDP-mannose biosynthesis pathway, the conversion of D-mannose 6-phosphate to D-mannose 1-phosphate. Amino acid sequence alignment indicated a high degree of identity to *P. aeruginosa* AlgC, which was shown to be bifunctional and exhibits phosphoglucomutase (PGM) as well as phosphomannomutase (PMM) activity [1], [25]. CBU0689 is annotated as GDP-mannose-4,6-dehydratase (GMD) and might provide the virenose biosynthetic intermediate GDP-6-deoxy-D-*lyxo*-hex-4-ulopyranose (GDP-Sug) by conversion of GDP-D-mannose. Further CBU0671 and CBU0689 are located within a genomic region associated with O-PS biosynthesis [26].

**Table 1:** Predicted *C. burnetii* proteins catalyzing formation of GDP-D-mannose.

Bacteria	Gene	Gene bank accession no.	% Identity/% Similarity	Putative function
***C. burnetii*** ** CBU0671**		AAO90215.1		Type II PMI
*E. coli* EDL933	*manA*	AAG56600.1	37/54	PMI
*E. coli* EDL933	*manC*	AAG57091.1	43/61	GMP
*P. aeruginosa* PAO1	*wbpW*	AAG08837.1	41/60	PMI/GMP
***C. burnetii*** ** CBU0294**		AAO89851.2		PMM
*P. aeruginosa* PAO1	*algC*	AAG08707.1	55/74	PMM/PGM
*E. coli* MS 175-1	PMM_PGM	EFJ67760	32/52	PMM/PGM
***C. burnetii*** ** CBU0689**		NP_819719		GMD
*E. coli* E110019	*gmd*	ZP_03050267	54/71	GMD

GMD, GDP-mannose-4,6-dehydratase; GMP, GDP-mannose pyrophosphorylase; PGM, phosphoglucomutase; PMI, phosphomannose isomerase; PMM, phosphomannomutase.

### *C. burnetii* CBU0671 exhibited GDP-mannose pyrophosphorylase (GMP) but not PMI activity

In order to characterize the enzymatic activities of *C. burnetii* CBU0671, this protein was expressed in its native form and used for complementation of *E. coli manA* and *manC* mutant strains, defective for O-PS or CPS synthesis, respectively. To test for PMI activity the CBU0671 containing plasmid pCN606_2 was introduced into the *manA* mutant strain *E. coli* CWG634 and O-PS patterns compared to wild type *E. coli* CWG28 O9a. Inactivation of *manA* in *E. coli* CWG364 was shown to abolish synthesis of mannose-6-phosphate, the precursor of GDP-D-mannose and resulted in a R-LPS phenotype [27]. Complementation of *E. coli* CWG634 with CBU0671 did not result in restoration of an S-LPS phenotype. Analysis of LPS from wild type, mutant and complemented strains using silver stained SDS-PAGE detected only revealed R-LPS chemotypes (data not shown). However, complementation of *E. coli* CWG634 with *E. coli* DH5α *manA* (pCN601a_5), which encoded a type I PMI, resulted in a smooth LPS phenotype (data not shown).

**Figure 2:**
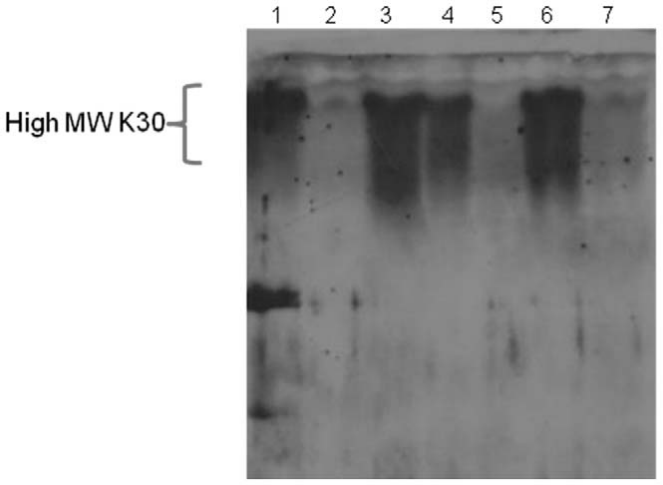
*C. burnetii* CBU0671 (GMP) restores K30 expression in the *E. coli cpsB* (*manC*) mutant strain CWG152. Immunoblot analysis with K30-specific antiserum of proteinase K treated whole cell lysates of 1. wild type *E. coli* CWG44, *E. coli cpsB* mutant strain CWG152, 3. *E. coli* CWG152/pCN603-1 (*E. coli cpsB*) induced, 4. *E. coli* CWG152/pCN603-1 (*E. coli cpsB*) not induced, 5. *E. coli* CWG152/pBAD (vector control), 6. *E. coli* CWG152/pCN606-2 (*C. burnetii* CBU0671) induced, 7. *E. coli* CWG152/pCN606-2 (*C. burnetii* CBU0671) not induced.

To test if CBU0671 exhibits GMP activity, plasmid pCN606_2 was introduced into the *E. coli manC* mutant strain CWG152 and the CPS pattern was compared to wild type *E. coli* CWG44 K30 [28], [29]. As a positive control, *manC* from *E. coli* DH5α was cloned and expressed in its native form and the resulting plasmid, pCN603_1, was introduced into CWG152. CPS isolated from wild-type strain *E. coli* CWG44, *manC* mutant strain *E. coli* CWG152 and complemented strains *E. coli* CWG152/pCN606-2 and *E. coli* CWG152/ pCN603_1 were analyzed using silver-stained SDS-PAGE and immunoblot with anti-K30 antiserum. Complementation of *E. coli* CWG152 with CBU0671 or *E. coli* DH5α *manC* resulted in typical high and low molecular mass CPS bands as detected for the wild type strain (Fig. 2). Taken together these data clearly demonstrated that CBU0671 exhibits GMP activity, but could not complement a type I PMI.

### *C. burnetii* CBU0294 exhibits PMM and PGM activity

**Figure 3:**
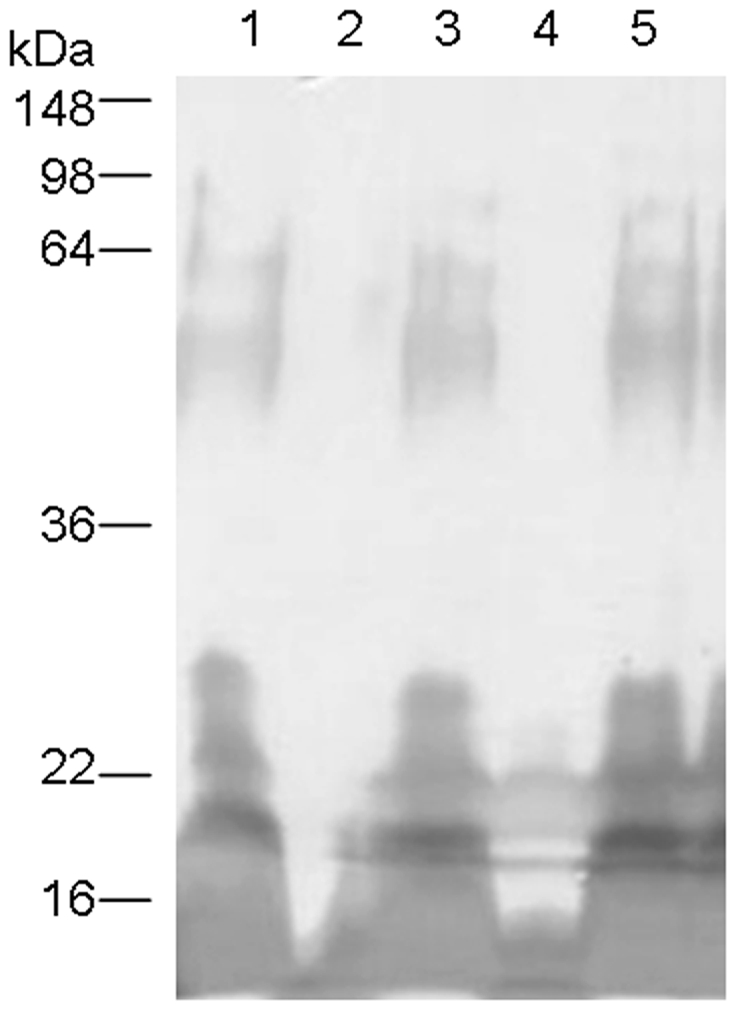
*C. burnetii* CBU0294 (PMM) restores a smooth LPS chemotype in *P. aeruginosa* PAO1 O5 *algC* mutant. SDS-PAGE and silver stain of proteinase K treated whole cell lysates of 1. wild-type *P. aeruginosa* PAO1, 2. *P. aeruginosa algC* mutant, 3. *P. aeruginosa algC::tet*/pLPS188 (*P. aeruginosa algC*), 4. *P. aeruginosa algC::tet*/pUCP20 (vector control), 5. *P. aeruginosa algC::tet*/pCN620.

The enzymatic function of *C. burnetii* CBU0294 was evaluated by complementation of an *algC* mutant of *P. aeruginosa* PAO1 serotype O5. PMM function of AlgC catalyzes the formation of mannose-1-phosphate, which is a metabolic precursor for synthesis of GDP-D-mannose [25]. The latter is converted to GDP-D-rhamnose, the sugar residue composing the O5 A-band homopolymer [30]. It has been shown that PGM function of *P. aeruginosa* AlgC is required for formation of D-glucose-1-phosphate, which is necessary for biosynthesis of UDP-D-glucose, a component of the core heterooligosaccharide [25], [31]. Therefore both, PMM and PGM functions of AlgC are required to visualize the *P. aeruginosa* PAO A-band. *C. burnetii* CBU0294 was cloned into the *P. aeruginosa* shuttle vector pUCP20 and the resulting plasmid, pCN620, used for transformation of *P. aeruginosa* PAO1 *algC::tet*. LPS samples prepared from transformed PAO1 *algC::tet* strains were separated by SDS-PAGE and visualized by silver staining (Fig. 3). Both, the parental and complemented mutant strains produced a typical LPS banding pattern, while PA01 *algC::tet* alone as well as the vector control did not produce A-band LPS.

To evaluate the PGM activity of *C. burnetii* CBU0294, pCN620 was transformed into *E. coli* W1485 *pgm::tet* and selected on MacConkey agar for the ability to metabolize galactose. *E. coli* W1485 *pgm::tet* that harbored pCN620 generated deep red colonies identical to *E. coli* W1485 wild type, whereas *E. coli* W1485 *pgm::tet* alone or the empty vector control *E. coli* W1485 *pgm::tet*/pUCP20 produced light pink colonies (data not shown). These data indicate that CBU0294 might encode a bifunctional enzyme, which exhibits PMM as well as PGM activity. Thus, CBU0294 is likely to catalyze the second step in the GDP-mannose biosynthesis pathway of *C. burnetii*.

### Determination of PMI, PMM and GMP activities of purified *C. burnetii* proteins

**Figure 4:**
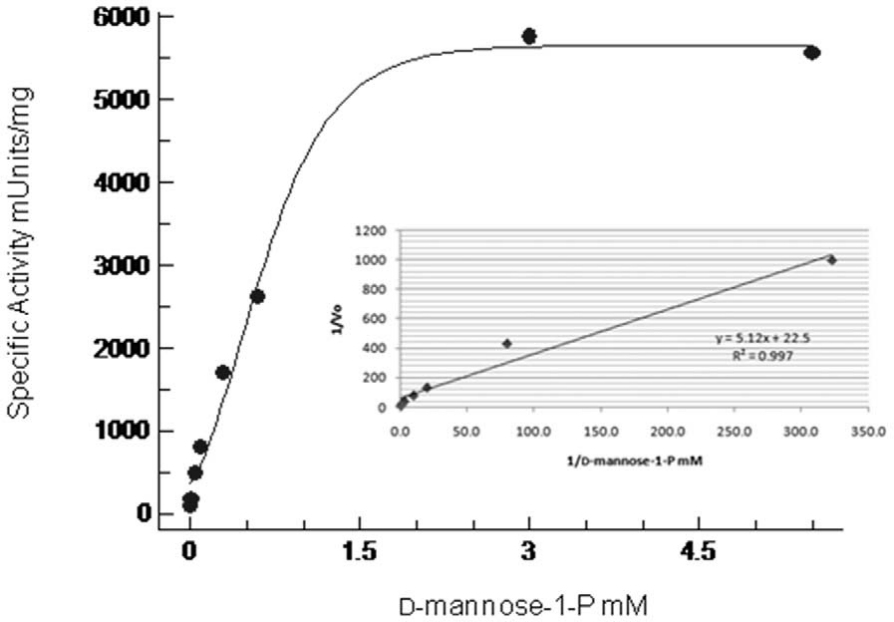
Mechaelis-Menten diagram depicting *C. burnetii* CBU0294 (PMM) kinetic parameters. Reactions were carried out with D-mannose-1-P as the fixed substrate. Data points were fitted using Microsoft XLfit model 601. Inset: Cooresponding Lineweaver-Burk Plot, regression line calculated by least squares.

*C. burnetii* CBU0671 and CBU0294 were expressed as His-tagged proteins in *E. coli* DH5α and isolated to near homogeneity. Kinetic analyses of purified enzymes were carried out by measuring initial enzyme specific activity relative to varying concentrations of substrate. Km and Vmax values were determined by Lineweaver-Burk Plot analysis with a regression coefficient greater than 0.99 (Fig. 4). Obtained Km and Vmax values as well as specific enzymatic activity for purified CBU0671 with mannose-1-phosphate or GDP-D-mannose as substrate indicate PMI and GMP activity (Table 2). PMI and GMP activity were also detectable in bacterial crude extracts comparable to *E. coli* ManA and ManC (Table 3). Specific enzymatic activity obtained for purified CBU0294 with mannose-1-phosphate indicates PMM activity, which is also detectable in crude extracts (Table 2 and 3).

**Table 2:** Kinetic parameters for *C. burnetii* CBU0671 and CBU0294.

CBU no.	Tested enzymatic activity	Substrate	Km [µmol L^−1^]	Vmax [µmol min^−1^]	Specific activity [mU/mg]
CBU0671	PMI	mannose-6-phosphate	11600	3.59	567
	GMP	GDP-D-mannose	379	0.757	97
CBU0294	PMM	mannose-1-phosphate	228	44	4174

GMP, GDP-mannose pyrophosphorylase; PMI, phosphomannose isomerase; PMM, phosphomannomutase.

**Table 3:** Enzymatic activity of *C. burnetii* CBU0671 and CBU0294 in bacterial crude extracts.

Tested enzymatic activity	*E. coli*	Specific activity [mU/mg]	*C. burnetii*	Specific activity [mU/mg]
PMI	ManA	6148	CBU0671	1971
PMM	ManB	ND	CBU0294	72
GMP	ManC	185	CBU0671	77.5

GMP, GDP-mannose pyrophosphorylase; PMI, phosphomannose isomerase; PMM, phosphomannomutase; ND, not detected.

GMD activity of *C. burnetii* CBU0689 was tested using the method described by Alberman et al. [32] by expression of the native protein in *E. coli*. Therefore *C. burnetii* CBU0689 was cloned into pBAD and the resulting plasmid, pCN608c-1, transformed *E. coli* DH5α. Enzyme activity was then measured directly in crude extracts by monitoring the increase in GDP-Sug at OD_320_ (ε_320 nm_ = 2.20 L⋅mmol^−1^⋅cm^−1^) in alkaline conditions. GMD activity for CBU0689 was determined as 14 NKat/mg. Taken together these data clearly show that all three *C. burnetii* open reading frames (*orf's*) exhibit the necessary enzymatic activities for formation of GDP-D-mannose and GDP-Sug as intermediates for virenose synthesis.

## Discussion

The goal of this work was to characterize the enzymatic steps responsible for formation of GDP-D-mannose in *C. burnetii*, which were bioinformatically predicted as the initial steps of GDP-β-D-virenose biosynthesis. Structural evidence of β-D-virenose isolated from the virulent phase I *C. burnetii* RSA493 O-PS further supports this hypothesis [19]. Although *C. burnetii* CBU0671, a predicted bifunctional type II PMI, failed to complement an *E. coli manA* mutant strain (PMI), it did complement an *E. coli manC* mutant strain (GMP). An exhaustive bioinformatic search of the annotated genome failed to reveal an alternative *C. burnetii* PMI. Clustal analysis showed that CBU0671 contained the signature sites observed in other type II PMIs, such as WbpW or AlgA of *P. aeruginosa*; pyrophosphorylase signature, GMP active site, nucleotidyl transferase domain, mannose-6-phosphate isomerase domain, zinc binding motif and PMI active site [23]. Althrough *C. burnetii* failed to complement a *manA* mutation in *E. coli in vitro* assays using natively formed and His-tagged *C. burnetii* CBU0671 showed specific activities, Km and Vmax values, comparable to previously reported values for PMI and GMP [33]. Differences in regulation or catalytic process might explain the observed distinct activities for CBU0671 in a *manA* deficient background or *in vitro* observed enzymatic activities, respectively. Both, type I and type II PMI's possess a highly conserved motif within the active side, but other proteins have lost the specific catalytic function despite the shared motif [24]. Further investigations, such as complementation of other type II PMI's are necessary to clearly identify the catalytic activities of CBU0671. Taken together, we report that CBU0671 is a new member of the small and poorly characterized class of proteins known as type II PMIs, based on complementation assays and biochemical characterization.

*C. burnetii* CBU0294 successfully complemented a *P. aeruginosa* O5 *algC* mutant strain and restored expression of a smooth LPS. Previous studies showed that *algC* of *P. aeruginosa* O-serotype O5 is involved in formation of D-mannose and D-glucose-1-phosphate, both necessary intermediates for synthesis of the O5 O-specific chain and core oligosaccharide. Therefore inactivation of *algC* leads to a deep rough phenotype in *P. aeruginosa* O5 [25], [30]. Restored expression of a smooth LPS by complementation indicates that *C. burnetii* CBU0294 simultaneously carried out PGM and PMM activities in this strain. To further demonstrate that *C. burnetii* CBU0294 also exhibits phosphoglucomutase activity, a *pgm E. coli* mutant strain, W1485 *pgm::tet*, was successfully complemented with CBU0294. This finding supports the notation *C. burnetii* CBU0294 is bifunctional and carries out PGM and PMM activities as described for *algC*.

Bioinformatic analysis indicats that CBU0689 encodes a GDP-mannose 4,6-dehydratase (GMD). When compared to *E. coli* GMD, CBU0689 was 52% identical, 69% similar on the amino acid level with an E_value_ of 5×10^−12^ [32], [34]. Specific activity of native *C. burnetii* GMD in crude extracts and its gene location within the LPS associated genome region in *C. burnetii* supports its bioinformatic assignment [26]. GDP-Sug formed by GMD is the metabolic intermediate of GDP-L-fucose, GDP-colitose, GDP-perosamine, GDP-D-rhamnose and GDP-6-deoxy-D-talose [35]. The enzymes required to generate the final steps required for GDP-perosamine (perosamine synthase CBU0830) and GDP-L-fucose (fucose synthase CBU0688) have been identified in the *C. burnetii* genome [26]. However, none of these activated saccharides have been observed in *C. burnetii* with the exception of a single report in which rhamnose was identified by GC-MS in the *C. burnetii* LPS outer core [12].

Since the characterization of the avirulent *C. burnetii* RSA439 genomic deletion [21], the enzymatic mechanism of fucose synthase, located within this region has become more clear [36]. Clustal analysis of the *C. burnetii* fucose synthase indicated that it bears the characteristic Ser-Tyr-Lys catalytic triad necessary to catalyze three reactions within a single active site; epimerization at both C3″ and C5″ and NADPH dependent reduction of the ketone at C4 [36]. Based on these data, the formation of GDP-β-D-virenose may ultimately be formed when GDP-L-fucose is modified by the addition of a methyl group at C3″ perhaps by CBU0691 and inversion of stereochemistry at the C2″ (Fig. S1).

A fundamental understanding of *C. burnetii* LPS biosynthesis and its structure are lacking. The intracellular nature of *C. burnetii*, lack of genetic tools and its status as a select agent has made elucidating these basic physiological mechanism challenging. This study establishes the foundation necessary to fully characterize the GDP-β-D-virenose biosynthesis pathway and ultimately the formation of *C. burnetii* O-PS, which is the only known virulence factor of *C. burnetii*.

## Materials and Methods

### Bacterial strains and growth conditions

Bacterial strains and plasmids used in this study are described in Table 4. All bacterial strains were routinely propagated at 37°C in Luria-Bertani (LB) broth or on LB-1.2% agar plates (Difco Laboratories). When necessary, ampicillin (100 µg/mL), carbenicillin (50 µg/mL), chloramphenicol (34 µg/mL), kanamycin (50 µg/mL), or tetracycline (12.5 µg/mL) was added to the media. *P. aeruginosa* strains were selected on carbenicillin (500 µg/mL) and tetracycline (100 µg/mL), as required.

**Table 4:** Bacterial strains and plasmids used in this study.

Strain	Characteristics	Reference
**Bacteria**		
*C. burnetii*	RSA 439, clone 4	[48]
*E. coli* DH5α	*F′(Φ80dΔ(lacZ)M15), recA1, endA1, gyrA96, thi1, hsdR17 (rk-mk+), supE44, relA1, deoR, Δ(lacZYA-argF), U169*	Stratagene
*E. coli* TOP 10	F^−^ *mcr*A Δ(*mrr*-*hsd*RMS-*mcr*BC) Φ80*lac*Z 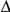 M15 Δ*lac*X74 *rec*A1 *ara*Δ139 Δ(*ara*-*leu*)7697 *gal*U *gal*K *rps*L (Str^R^) *end*A1 *nup*G	Invitrogen
*E. coli* CWG 28	*Trp his lac rpsL cpsK*30^−1^ (Sm^r^ O9a:K30^−^)	[27]
*E. coli* CWG 634	*Trp his lac rpsL cpsK*30^−1^ *manA4* (Sm^r^ Tc^r^ O9a^−^:K30^−^)	[27]
*E. coli* CWG 44	*his trp lac rpsL* (09-:K30:H12; rfb09)	[28]
*E. coli* CWG 152	CWG44 but O-:K-:*H12rfbM*	[28]
*E. coli* W1485	Wild type *E. coli*	[25]
*E. coli* W1485 *pgm*:tet	*pgm* mutant of W1485	[25]
*P. aeruginosa* PAO1	Serotype O5	[25]
*P. aeruginosa* PAO1 *algC*::tet	*algC* mutant of PAO1 (LPS O5^−^)	[25]
**Plasmids**		
pBAD	Expression vector, Amp^R^	Invitrogen
pUCP20	*P. aeruginosa* shuttle vector, Carb^R^	[44]
pLPS188	pUCP18, *P. aeruginosa algC*	[25]
pCN601a_5	pBAD, E. *coli* DH5α *manA*, native	This study
pCN601c_A1	pBAD, E. *coli* DH5α *manA*, poly-His	This study
pCN603_1	pBAD, *E. coli* DH5α *cpsB* (*manC*), native	This study
pCN603a_A4	pBAD, *E. coli* DH5α *cpsB* (*manC*), poly-His	This study
pCN606_2	pBAD, CBU0671, native	This study
pCN606c_E1	pBAD, CBU0671, poly-His	This study
pCN607a_3	pBAD, CBU0294, native	This study
pCN607z_A2	pBAD, CBU0294, poly-His	This study
pCN608c_1	pBAD, CBU0689, native	This study
pCN620	pUCP20, CBU0294	This study

### General DNA methods

DNA isolation and manipulations were carried out in according to Sambrook and Russel (2001) [37]. Oligonucleotides used in this study are listed in Table S1. DNA restriction endonucleases, T4 DNA ligase and Accuprime polymerase (Invitrogen) were used as advised by the manufacturer. Electrocompetent *E. coli* and *P. aeruginosa* cells were prepared as described elsewhere [38], [39] and transformed using a Bio-Rad Gene-Pulser Transfection Apparatus (200Ω, 25 µF, 12.5 kV/cm, 4.7 ms).

### Complementation of *E. coli manA* and *manC* mutant strains with *C. burnetii* CBU0671

CBU071, including the native stop codon, was amplified from chromosomal DNA of *C. burnetii* RSA 439 with CBU0672FNcoI and CBU0671R and cloned into pBAD for native protein expression. The resulting plasmid, pCN606_2, was used for complementation LPS *manA* or CPS *manC E. coli* mutant strains CWG634 and CWG152. As positive controls *E. coli* DH5α *manA* (EcmanAF/EcmanAR) and *manC* (EccpsBNcoIF/EccpsBR) were cloned into pBAD and the resulting plasmids, pCN601a_5 and pCN603_1, used for native protein expression in the corresponding *manA* and *manB* mutant strains. All generated plasmids were verified by sequencing. Complemented strains were grown in LB broth supplemented with 0.4% glucose, to avoid uptake of exogenous mannose, and protein expression induced with 0.2% arabinose over night [27]. Expression of full length LPS or CPS was determined by analysis of proteinase K-treated whole cell lysates of complemented strains and compared to corresponding LPS and CPS wild type strains *E. coli* CWG28 (serotype O9a) and *E. coli* CWG44 (serotype K30), respectively. Lysates were prepared as described elsewhere [40] and analyzed by sodium dodecyl sulphate polyacrylamide gel electrophoresis (SDS-PAGE) and stained with silver nitrate or transferred to nitrocellulose membrane (Bio-RAD) [40], [41], [42], [43]. O9a (1∶2000) or K30-specific antiserum (1∶1000) was used for detection of LPS and CPS expression and traced with horse radish peroxidase-conjugated goat anti-rabbit IgG(γ) monoclonal antibody (1∶5000) with peroxidase substrate in according to the guidelines of the manufacturer (Amersham Bioscience).

### Complementation of *P. aeruginosa algC* mutant strain with *C. burnetii* CBU0294

CBU0294 was amplified (CBU0294FScaI/CBU0294RXbaI) from chromosomal *C. burnetii* RSA 439 DNA and obtained DNA fragment was digested with *Sca*I and *Xba*I. CBU0294 was subsequently cloned into the *Sca*I, *Xba*I treated shuttle vector pUCP20 to generate pCN620 [44]. Correct insertion of CBU0294 in pCN620 was verified by sequencing. For complementation studies *P aeruginosa* PAO1 *algC*:tet was transformed with pCN620 and additionally with a *P. aeruginosa algC* containing shuttle vector, pLPS188 [25]. LPS banding patterns from *P aeruginosa* PAO1 *algC*:tet harboring pCN620 or pLPS188 were analyzed as described under 4.3 and compared to wild type LPS from *P.aeruginosa* PAO1. PAO1 specific antiserum (1∶1000) was used for detection of OPS expression and traced with horse radish peroxidase-conjugated goat anti-rabbit IgG(γ) monoclonal antibody (1∶5000).

### Phosphoglucomutase (*pgm*) complementation of *E. coli* with *C. burnetii* CBU0294

CBU0294 was amplified (CBU0294F/CBU0294R) and cloned into pBAD for native protein expression. The resulting plasmid, pCN607a_3, was sequenced and used for transformation of *E. coli* W1485*pgm::tet*. Phosphoglucomutase positive wild type *E. coli* W1485 and complemented mutant strains, were then distinguished from *pgm* negative strains using MacConkey agar (Difco Laboratories) supplemented with 1% (w/v) galactose and 0.2% (w/v) arabinose as previously described [45].

### Cloning and expression of *C. burnetii* GDP-D-mannose synthesis genes for enzyme activity assays

*C. burnetii* CBU0671 (CBU0671F/CBU0671Rpoly-His) and CBU0294 (CBU0294Fpoly-His/CBU0294Rpoly-His) were amplified and subsequently cloned into pBAD for expression of His-tagged proteins. The generated plasmids pCN606c_E1 and pCN607z_A2 were sequenced for correct insertion of target genes and used for transformation of *E. coli* DH5α. As controls *E. coli* DH5α *manA* and *cpsB* (*manC*) genes were amplified with EcmanAFpoly-His and EcmanARpoly-His or EccpsBFpoly-His and EccpsBFpoly-His and cloned into pBAD. The resulting plasmids pCN601c_A1 and pCN603a_A4 were sequenced for correct insertion of target genes and used for transformation of *E. coli* DH5α. Expression was induced with 0.2% arabinose for 4 to 8 h at an OD_600_ of 0.6. Bacteria were harvested (10,000×g, 10 min, 4°C), resuspended in 10 mL binding buffer (25 mM NaPO_4_, 0.5 M NaCl, 10 mM imidazole, pH 8.0) with DNase (10 µg/mL), RNase (10 µg/mL) and lysozyme (10 µg/mL) and incubated for 30 minutes on ice. Cells were lysed using French press and cell debris separated by centrifugation (24,400×g, 60 min, 4°C). His-tagged proteins were isolated from supernatants (crudes extracts) using the ProBond purification system as described by the supplier (Invitrogen). Purified proteins were analyzed for purity and size by SDS-PAGE and silver staining or immunoblot analysis with 6×His monoclonal antibody (1∶5000, Clontech). PMI, PMM or GMD activity was also determined in bacterial crude extracts that contained natively formed *C. burnetii* proteins.

### Phosphomannose isomerase (PMI) in vitro assay

PMI enzyme activity was determined by monitoring the reduction of NADP^+^ at 340 nm (ε_M_ = 6.22 mM^−1^ cm^−1^) [46]. One unit of enzyme activity was defined as the detection of 1 µmole of product per minute. Concentration of purified enzyme was determined using the Micro BCA Protein Assay (Invitrogen) as described by the supplier. PMI activity was determined by a modified protocol described by Sa-Correia *et al.* [33]. The reaction mixture in a 1 mL total volume contained 10 µmol of MgCl_2_, 1.0 µmol of NADP^+^, 1 unit phosphoglucose isomerase, 1 unit glucose-6-phoshphate dehydrogenase, 1.1 µmol of D-mannose-6-phosphate in 50 mM tris HCl buffer pH 7.55. MgCl_2_, NADP^+^ and D-mannose-6-phosphate were dissolved in 50 mM tris HCl buffer pH 7.55 prior to adding them to the reaction mixture. The reaction mixture was equilibrated for 5 min at 25°C and the reaction initiated by adding 50 to 200 µL of crude extract that contained natively formed PMI or 7–15 µg of purified His-tagged PMI (CBU0671).

### Phosphomannomutase (PMM) in vitro assay

PMM activity was determined by monitoring the reduction of NADP^+^ at 340 nm (4.6.1.) as described by Sa-Correia *et al.* [33]. The reaction mixture in a 1 mL total volume contained 10 µmol of MgCl_2_, 1.0 µmol of NADP^+^, 1 unit phosphoglucose isomerase, 1 unit glucose-6-phoshphate dehydrogenase, 5 units of purchased PMI (Sigma), 0.25 µmol of D-glucose-1,6-diphosphate (ADGD) and 5.5 µmol of D-mannose-1-phosphate in 50 mM tris HCl buffer pH 7.55. *C. burnetii* CBU0294 is annotated as a bifunctional phosphomannomutase (PMM) and phosphoglucomutase (PGM). Therefore, the addition of ADGD moved the kinetics of the reaction towards the formation of D-gluconate-6-phosphate. The reaction mixture was equilibrated for 5 minutes at 25°C and the reaction initiated by adding 50–200 µl of crude extract that contained natively formed PMM or of 7–15 µg of purified His-tagged PMM (CBU0294).

### GDP-D-mannose pyrophosphorylase (GMP) in vitro assay

GMD activity was determined using a modified protocol described by Munch-Peterson *et al.* [47], monitoring the reduction of NADP^+^ (4.6.1.). The reaction mixture in a 1 mL total volume contained 10 µmol of MgCl_2_, 1.0 µmol of NADP^+^, 0.1 µmol of ADP, 2 µmol of PPi, 5.0 µmol of NaF, 1 unit of hexokinase, 1 unit of nucleoside kinase, 1 unit of glucose-6-phoshphate dehydrogenase, 0.8 µmol of glucose, 5.5 µmol of GDP-D-mannose in 50 mM tris HCl buffer pH 7.55. The reaction mixture was equilibrated for 5 minutes at 25°C, and initiated by adding 50–200 µl of crude extract. Endogenous activity in crude extracts of *E. coli* DH5α carrying the empty pBAD vector were subtracted from the test samples.

### GDP-D-mannose 4,6-dehydratase (GMD) in vitro assay

CBU0689 was amplified (CBU0689FNcoI/CBU0689R) and cloned into pBAD, retaining the native stop codon. The resulting plasmid, pCN608c_1, was used for transformation of *E. coli* DH5α. Crude extract GMD activity was determined by a modified protocol described by Albermann *et al.* [32]. The reaction mixture in a total volume of 300 µL contained 10 µmol of MgCl2, 1.0 µmol of NADP^+^ and 5.5 µmol of GDP-D-mannose in 50 mM tris HCl buffer pH 7.55. After equilibrating for 5 minutes at 37°C, the reaction was initiated by adding 60 µL of prewarmed crude extract. Aliquots of 50 µL were taken every 10 min and added to 950 µL of 37°C 100 mM NaOH. The reaction was incubated for an additional 20 minutes. The formation of GDP- 4-keto-6-deoxy-D-mannose was measured directly at OD_320_ (ε_M_ = 2.2 mM^−1^ cm^−1^) [34].

## Supporting Information

Figure S1 **Clustal analysis of** ***C. burnetii*** **fucose synthase CBU0688 (GFS).** The *C. burnetii* GFS has the characteristic “Catalytic Triad,” Ser (S) 107-Tyr (Y) 136-Lys (K) 140 boxed in black, observed in SDR family enzymes. Additionally, boxed in red are active sites implicated as the acid/bases involved in promoting the epimerization reactions.
(TIF)

Table S1 **Oligonucleotides used in this study.** *Introduced endonuclease restriction sites are underlined.
(DOC)

## References

[1] Raetz CR, Whitfield C (2002). Lipopolysaccharide endotoxins.. Annu Rev Biochem.

[2] Madariaga MG, Rezai K, Trenholme GM, Weinstein RA (2003). Q fever: a biological weapon in your backyard.. Lancet Infect Dis.

[3] Zhang G, Samuel JE (2004). Vaccines against Coxiella infection.. Expert Rev Vaccines.

[4] Stoker MG, Fiset P (1956). Phase variation of the Nine Mile and other strains of *Rickettsia burnetii*.. Can J Microbiol.

[5] Hackstadt T (1988). Steric hindrance of antibody binding to surface proteins of *Coxiella burnetii* by phase I lipopolysaccharide.. Infect Immun.

[6] Lukacova M, Kazar J, Gajdosova E (1994). *Coxiella burnetii* phase I and II proteins studied by SDS-page.. Acta Virol.

[7] Moos A, Hackstadt T (1987). Comparative virulence of intra- and interstrain lipopolysaccharide variants of *Coxiella burnetii* in the guinea pig model.. Infect Immun.

[8] Zhang G, Russell-Lodrigue KE, Andoh M, Zhang Y, Hendrix LR (2007). Mechanisms of vaccine-induced protective immunity against *Coxiella burnetii* infection in BALB/c mice.. J Immunol.

[9] Toman R, Hussein A, Palkovic P, Ftacek P (2003). Structural properties of lipopolysaccharides from *Coxiella burnetii* strains Henzerling and S.. Ann N Y Acad Sci.

[10] Schramek S, Mayer H (1982). Different sugar compositions of lipopolysaccharides isolated from phase I and pure phase II cells of *Coxiella burnetii*.. Infect Immun.

[11] Amano K, Williams JC, Missler SR, Reinhold VN (1987). Structure and biological relationships of *Coxiella burnetii* lipopolysaccharides.. J Biol Chem.

[12] Toman R, Kazar J (1991). Evidence for the structural heterogeneity of the polysaccharide component of *Coxiella burnetii* strain Nine Mile lipopolysaccharide.. Acta Virol.

[13] Toman R, Skultety L (1996). Structural study on a lipopolysaccharide from *Coxiella burnetii* strain Nine Mile in avirulent phase II.. Carbohydr Res.

[14] Skultety L, Toman R, Pätoprsty V (1998). A comparative study of lipopolysaccharides from two *Coxiella burnetii* strains considered to be associated with acute and chronic Q fever.. Carbohydrate Polymers.

[15] Ftacek P, Skultety L, Toman R (2000). Phase variation of *Coxiella burnetii* strain Priscilla: influence of this phenomenon on biochemical features of its lipopolysaccharide.. J Endotoxin Res.

[16] Toman R, Skultety L, Kazar J (1993). On the determination of “Kdo-like substance” in the lipopolysaccharide from *Coxiella burnetii* strain nine mile in phase II.. Acta Virol.

[17] Toman R, Garidel P, Andra J, Slaba K, Hussein A (2004). Physicochemical characterization of the endotoxins from *Coxiella burnetii* strain Priscilla in relation to their bioactivities.. BMC Biochem.

[18] Schramek S, Radziejewska-Lebrecht J, Mayer H (1985). 3-C-branched aldoses in lipopolysaccharide of phase I *Coxiella burnetii* and their role as immunodominant factors.. Eur J Biochem.

[19] Toman R, Skultety L, Ftacek P, Hricovini M (1998). NMR study of virenose and dihydrohydroxystreptose isolated from *Coxiella burnetii* phase I lipopolysaccharide.. Carbohydr Res.

[20] Vadovic P, Slaba K, Fodorova M, Skultety L, Toman R (2005). Structural and functional characterization of the glycan antigens involved in immunobiology of Q fever.. Ann N Y Acad Sci.

[21] Hoover TA, Culp DW, Vodkin MH, Williams JC, Thompson HA (2002). Chromosomal DNA deletions explain phenotypic characteristics of two antigenic variants, phase II and RSA 514 (crazy), of the *Coxiella burnetii* nine mile strain.. Infect Immun.

[22] Denison AM, Massung RF, Thompson HA (2007). Analysis of the O-antigen biosynthesis regions of phase II Isolates of *Coxiella burnetii*.. FEMS Microbiol Lett.

[23] Sousa SA, Moreira LM, Leitao JH (2008). Functional analysis of the *Burkholderia cenocepacia* J2315 BceAJ protein with phosphomannose isomerase and GDP-D-mannose pyrophosphorylase activities.. Appl Microbiol Biotechnol.

[24] Jensen SO, Reeves PR (1998). Domain organisation in phosphomannose isomerases (types I and II).. Biochim Biophys Acta.

[25] Omsland A, Cockrell DC, Fischer ER, Heinzen RA (2008). Sustained axenic metabolic activity by the obligate intracellular bacterium *Coxiella burnetii*.. J Bacteriol.

[26] Seshadri R, Paulsen IT, Eisen JA, Read TD, Nelson KE (2003). Complete genome sequence of the Q-fever pathogen *Coxiella burnetii*.. Proc Natl Acad Sci U S A.

[27] Clarke BR, Cuthbertson L, Whitfield C (2004). Nonreducing terminal modifications determine the chain length of polymannose O antigens of *Escherichia coli* and couple chain termination to polymer export via an ATP-binding cassette transporter.. J Biol Chem.

[28] Jayaratne P, Bronner D, MacLachlan PR, Dodgson C, Kido N (1994). Cloning and analysis of duplicated *rfbM* and *rfbK* genes involved in the formation of GDP-mannose in *Escherichia coli* O9:K30 and participation of *rfb* genes in the synthesis of the group I K30 capsular polysaccharide.. J Bacteriol.

[29] Whitfield C (2006). Biosynthesis and assembly of capsular polysaccharides in *Escherichia coli*.. Annu Rev Biochem.

[30] King JD, Kocincova D, Westman EL, Lam JS (2009). Review: Lipopolysaccharide biosynthesis in *Pseudomonas aeruginosa*.. Innate Immun.

[31] Darwin KH, Nathan CF (2005). Role for nucleotide excision repair in virulence of *Mycobacterium tuberculosis*.. Infect Immun.

[32] Albermann C, Piepersberg W (2001). Expression and identification of the RfbE protein from *Vibrio cholerae* O1 and its use for the enzymatic synthesis of GDP-D-perosamine.. Glycobiology.

[33] Sa-Correia I, Darzins A, Wang SK, Berry A, Chakrabarty AM (1987). Alginate biosynthetic enzymes in mucoid and nonmucoid *Pseudomonas aeruginosa*: overproduction of phosphomannose isomerase, phosphomannomutase, and GDP-mannose pyrophosphorylase by overexpression of the phosphomannose isomerase (*pmi*) gene.. J Bacteriol.

[34] Albermann C, Distler J, Piepersberg W (2000). Preparative synthesis of GDP-beta-L-fucose by recombinant enzymes from enterobacterial sources.. Glycobiology.

[35] Samuel G, Reeves P (2003). Biosynthesis of O-antigens: genes and pathways involved in nucleotide sugar precursor synthesis and O-antigen assembly.. Carbohydr Res.

[36] Lau ST, Tanner ME (2008). Mechanism and active site residues of GDP-fucose synthase.. J Am Chem Soc.

[37] Sambrook J, Russell D (2001). Molecular Cloning: A Laboratory Manual.

[38] Choi KH, Kumar A, Schweizer HP (2006). A 10-min method for preparation of highly electrocompetent *Pseudomonas aeruginosa* cells: application for DNA fragment transfer between chromosomes and plasmid transformation.. J Microbiol Methods.

[39] Dower WJ, Miller JF, Ragsdale CW (1988). High efficiency transformation of *E. coli* by high voltage electroporation.. Nucleic Acids Res.

[40] Hitchcock PJ, Brown TM (1983). Morphological heterogeneity among *Salmonella* lipopolysaccharide chemotypes in silver-stained polyacrylamide gels.. J Bacteriol.

[41] Laemmli UK (1970). Cleavage of structural proteins during the assembly of the head of bacteriophage T4.. Nature.

[42] Towbin H, Staehelin T, Gordon J (1979). Electrophoretic transfer of proteins from polyacrylamide gels to nitrocellulose sheets: procedure and some applications.. Proc Natl Acad Sci U S A.

[43] Tsai CM, Frasch CE (1982). A sensitive silver stain for detecting lipopolysaccharides in polyacrylamide gels.. Anal Biochem.

[44] West SE, Schweizer HP, Dall C, Sample AK, Runyen-Janecky LJ (1994). Construction of improved *Escherichia*-*Pseudomonas* shuttle vectors derived from pUC18/19 and sequence of the region required for their replication in *Pseudomonas aeruginosa*.. Gene.

[45] Adhya S, Schwartz M (1971). Phosphoglucomutase mutants of *Escherichia coli* K-12.. J Bacteriol.

[46] Gracy RW, Noltmann EA (1968). Studies on phosphomannose isomerase. I. Isolation, homogeneity measurements, and determination of some physical properties.. J Biol Chem.

[47] Munch-Peterson A (1962). GDP-mannose pyrophosphorylase.. Methods Enzymology.

[48] Samuel JE, Frazier ME, Mallavia LP (1985). Correlation of plasmid type and disease caused by *Coxiella burnetii*.. Infect Immun.

