# Supplementary figures and images for: Characterization of the GDP-D-Mannose Biosynthesis Pathway in Coxiella burnetii: The Initial Steps for GDP-β-D-Virenose Biosynthesis
Source: PLoS One. 2011 Oct 31;6(10):e25514. doi: 10.1371/journal.pone.0025514 (PMC3204966; doi:10.1371/journal.pone.0025514)

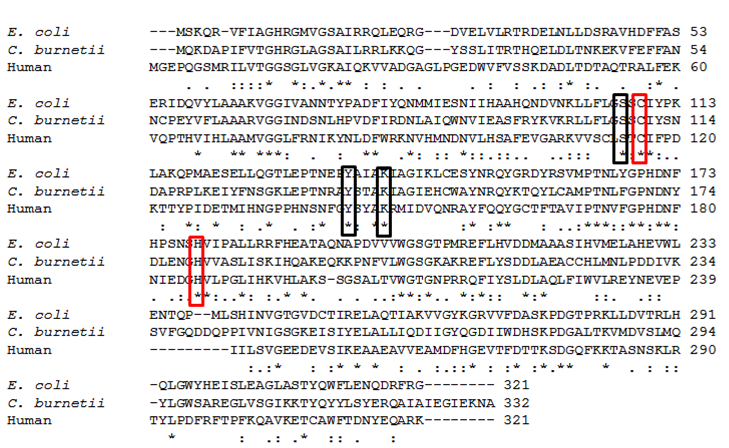

Supplement: Figure S1 — Clustal analysis of C. burnetii fucose synthase CBU0688 (GFS). The C. burnetii GFS has the characteristic “Catalytic Triad,” Ser (S) 107-Tyr (Y) 136-Lys (K) 140 boxed in black, observed in SDR family enzymes. Additionally, boxed in red are active sites implicated as the acid/bases involved in promoting the epimerization reactions. (TIF) [file pone.0025514.s001.tif]
