# Supplementary material for: Characterization of the GDP-D-Mannose Biosynthesis Pathway in Coxiella burnetii: The Initial Steps for GDP-β-D-Virenose Biosynthesis
Source: PLoS One. 2011 Oct 31;6(10):e25514. doi: 10.1371/journal.pone.0025514 (PMC3204966; doi:10.1371/journal.pone.0025514)
Supplement: Table S1 — Oligonucleotides used in this study. *Introduced endonuclease restriction sites are underlined. (DOC) [file pone.0025514.s002.doc]

| **Primer Name** | **Sequence*** | **Description** |
| --- | --- | --- |
| pBAD Forward | 5’-ATGCCATAGCATTTTTATCC-3’ | Sequencing primer |
| pBAD Reverse | 5’-GATTTAATCTGTATCAGG-3’ | Sequencing primer |
| EcmanAF | 5’-ATCATGCAAAAACTCATTAACTCAGTGCAAAACT-3’ | Expression of native protein |
| EcmanAR | 5’-GGGTTTCAATCTTCTTGCCTGTATACCATGC-3’ |
| EcmanAFpoly-His | 5’-ATGCAAAAACTCATTAACTCAGTGC-3’ | Expression of His-tagged protein |
| EcmanARpoly-His | 5’-GTTAATTTTTTCAGTAAGCTCTCCCAGC-3’ |
| EccpsBFNcoI | 5’-ATTCGGGGATAACCATGGCGCAGTC-3’ | Expression of native protein |
| EccpsBR | 5’-CAACGCCCGCATCCTGTAAACCT-3’ |
| EccpsBFpoly-His | 5’-ATTATGGCGCAGTCGAAACTCTATCCAGTTGTG-3’ | Expression of His-tagged protein |
| EccpsBFpoly-His | 5’-CGTTCCCACCCGTCCGTAGCGA-3’ |
| CBU0671FNcoI | 5’-GAACAGGATAACACCATGGAAGAGT-3’ | Expression of native protein |
| CBU0671R | 5’-TGAGTCACATTATTTCCAGCAGTT-3’ |
| CBU0671Fpoly-His | 5’-ATGGAAGAGTGCATAGTTCCAGTTTTGCTCGC-3’ | Expression of His-tagged protein |
| CBU0671Rpoly-His | 5’-GATGCGCCAATCGCCCCTTCCCAAC-3’ |
| CBU0294F | 5’-GTGCCGGCGACGCTTTTTAGAGC-3’ | Expression of native protein |
| CBU0294R | 5’-TAGTAAACTGGTTGACCGCACGAGAAAATC-3’ |
| CBU0294Fpoly-His | 5’-CAATGGAATGTCCCCGAGATAGTG-3’ | Expression of His-tagged protein |
| CBU0294Rpoly-His | 5’-AAAGGGCAACTCCAACGCATTATCA-3’ |
| CBU0689FNcoI | 5’-TGCAATTGAGGGAATAGAGAACCATGGG-3’ | Expression of native protein |
| CBU0689R | 5’-CGATTTCAACCGTGCCATCAGTGCTT-3’ |
| pUCP20F | 5’-TAACGCCAGGGTTTTCCCAGTCA-3’ | Sequencing primer |
| pUCP20R | 5’-TATGCTTCCGGCTCGTATGTTGT-3’ | Sequencing primer |
| CBU0294FScaI | 5’-TAGAGCTCTTTGAACTTACCGAACGAGGAGC-3’ |  |
| CBU0294RXbaI | TATCTAGATAGTAAACTGGTTGACCGCACGA-3’ |  |
